# Two Nested Inversions in the X Chromosome Differentiate the Dominant Malaria Vectors in Europe, *Anopheles atroparvus* and *Anopheles messeae*

**DOI:** 10.3390/insects15050312

**Published:** 2024-04-26

**Authors:** Evgenia S. Soboleva, Kirill M. Kirilenko, Valentina S. Fedorova, Alina A. Kokhanenko, Gleb N. Artemov, Igor V. Sharakhov

**Affiliations:** 1 Laboratory of Ecology, Genetics and Environmental Protection, Tomsk State University, 36 Lenin Avenue, Tomsk 634050, Russia; 2 Department of Entomology, the Fralin Life Sciences Institute, Virginia Polytechnic Institute and State University, 360 West Campus Drive, Blacksburg, VA 24061, USA

**Keywords:** chromosome inversions, X chromosome, breakpoint regions, synteny blocks, malaria mosquitoes, *Anopheles*

## Abstract

**Simple Summary:**

*Anopheles* mosquitoes are the only vectors of human malaria, a deadly disease causing over 500,000 deaths annually, mainly in sub-Saharan Africa. Less than 10% of the roughly 500 *Anopheles* species are malaria vectors. Although malaria has been eliminated in Europe, *Anopheles* mosquito species remain a focus of research due to their potential to transmit malaria and other infectious diseases. Understanding the evolution of vectorial capacity can be enhanced by knowledge of the speciation and adaptation of malaria mosquitoes. Chromosomal inversions, such as large-scale genomic rearrangements, are believed to play a role in the ability of mosquitoes to diversify and adapt to human-created environments. However, genome mapping and the characterization of chromosomal inversions have only been conducted on a small fraction of malaria mosquito species. In this study, we mapped and characterized inversions that differentiate the European mosquito *Anopheles atroparvus* and the Eurasian mosquito *An. messeae*. Two small genomic blocks underwent a change in position and orientation on the X chromosome between the two species. The rearranged chromosomal regions are enriched with genes that play roles in regulating the immune system and mating behavior. These findings offer insight into the underlying molecular mechanisms behind the differences in susceptibility to infection and behavior between *An. atroparvus* and *An. messeae*.

**Abstract:**

The Maculipennis subgroup of malaria mosquitoes includes both dominant malaria vectors and non-vectors in Eurasia. Understanding the genetic factors, particularly chromosomal inversions, that differentiate *Anopheles* species can provide valuable insights for vector control strategies. Although autosomal inversions between the species in this subgroup have been characterized based on the chromosomal banding patterns, the number and positions of rearrangements in the X chromosome remain unclear due to the divergent banding patterns. Here, we identified two large X chromosomal inversions, approximately 13 Mb and 10 Mb in size, using fluorescence in situ hybridization. The inversion breakpoint regions were mapped by hybridizing 53 gene markers with polytene chromosomes of *An. messeae*. The DNA probes were designed based on gene sequences from the annotated *An. atroparvus* genome. The two nested inversions resulted in five syntenic blocks. Only two small syntenic blocks, which encompass 181 annotated genes in the *An. atroparvus* genome, changed their position and orientation in the X chromosome. The analysis of the *An. atroparvus* genome revealed an enrichment of gene ontology terms associated with immune system and mating behavior in the rearranged syntenic blocks. Additionally, the enrichment of DNA transposons was found in sequences homologous to three of the four breakpoint regions. This study demonstrates the successful application of the physical genome mapping approach to identify rearrangements that differentiate species in insects with polytene chromosomes.

## 1. Introduction

Chromosomal inversions are drivers of genome evolution that play a role in adaptation and speciation of diploid organisms [1,2]. A reverse order of the genetic material in the chromosome due to an inversion causes the suppression of recombination in a heterozygous organism during meiosis that leads to the accumulation of divergent alleles. As a result, alternative arrangements of polymorphic inversions may contribute to the diversification of populations by providing ecological, behavioral, and physiological adaptations to changing environments and may finally lead to speciation. Polymorphic inversions in malaria mosquitoes have been shown to be associated with epidemiologically important traits such as adaptation to human-made habitats, host-seeking and blood-feeding behavior, and susceptibility to *Plasmodium* [3,4]. Fixed chromosomal inversions between the species are used by researchers to reconstruct species phylogenies as an independent approach to molecular phylogeny. For example, chromosomal phylogeny has identified ancestral and derived karyotypes in malaria mosquitoes of the *Anopheles gambiae* complex [4,5,6,7]. A recent combination of the whole-genome and inversion-based approaches reconstructed the phylogeny of mosquito species of the Maculipennis subgroup [8].

The *Anopheles* Maculipennis group comprises 24 species that inhabit the Holarctic zone and are classified into three subgroups: Freeborni, Quadrimaculatus, and Maculipennis [9]. The Maculipennis subgroup comprises species of malaria mosquitoes found in northern Eurasia. According to a recent multi-gene phylogeny reconstruction, supported by cytogenetic analysis, the ancestral species of these mosquitoes migrated from North America to Eurasia through Bering Land Bridge about 20 million years ago and were later divided into three clades: the Southern Eurasia clade, the European clade, and the Northern Eurasian clade [8]. *Anopheles atroparvus* is the dominant vector in much of Europe and to a lesser extent in the western part of Russia [10,11]. *Anopheles messeae* is the most widespread species that belongs to the Northern Eurasian clade of the Maculipennis subgroup. Although malaria was eliminated from the area of *An. messeae* distribution more than half of the century ago, this dominant malaria vector is still considered a potential public health threat [9]. The distribution of *An. messeae* extends from Ireland in the West to the Amur River region in the East and from Scandinavia and Yakutia in the North to Iran and Northern China in the South [12,13,14,15]. *Anopheles messeae,* along with other members of the Northern Eurasian clade, *An. daciae* and *An. maculipennis*, survive harsh winters by entering a complete diapause. The ability of *An. messeae* to occupy diverse ecological zones may be explained by a well-developed chromosomal inversion polymorphism. There are five wide-spread highly polymorphic inversions located on four chromosome arms: X1 and X2 on chromosome X; 2R1 on chromosome 2; 3R1 and 3L1 on different arms of chromosome 3 [16,17]. Also, a number of fixed chromosomal inversions differentiate genomes of the Maculipennis subgroup species. Approximate locations of breakpoints of the fixed inversions in the Maculipennis subgroup have been identified by reading the banding patterns of polytene chromosomes. For example, cytogenetic studies identified shared X chromosomal and autosomal arrangements between the members of the European clade *An. atroparvus* and *An. labranchiae*, but they had fixed autosomal inversions in comparison with *An. maculipennis*. Although fixed rearrangements on the X chromosome have been observed between *An. atroparvus* and *An. messeae* [18,19], their number and location could not be determined using traditional cytogenetics due to the divergent chromosomal banding patterns between the species. The precise mapping and characterization of fixed inversions and their breakpoints in mosquito genomes can be useful for the better understanding of mechanisms of rearrangements and the identification of genes involved in species differentiation.

Chromosomal inversions can contribute to species divergence [20]. Therefore, it is important to study the biological roles of genes located inside inversions, as well as to analyze repetitive DNA sequences in the breakpoint regions to comprehend how the inversion functions and originates [21,22]. The genome of *An. atroparvus* is the available reference assembly that can be used for comparative physical mapping with other members of the Maculipennis subgroup [23,24].

In this study, we performed physical genome mapping and molecular characterization of the X chromosomal regions involved in rearrangements between the malaria mosquito species *An. messeae* and *An. atroparvus*. We identified and characterized conserved synteny blocks (SBs) and breakpoint regions (BRs) of the inversions using the genome of *An. atroparvus*. We also determined the ancestral and derived X chromosomal arrangements in the Maculipennis subgroup.

## 2. Materials and Methods

### 2.1. Mosquito Collection, Species Identification, and Karyotyping

*Anopheles* 4th-instar larvae were collected from a natural pond in the town of Togur (Tomsk region, Russia, 58°22′12.7″ N; 82°51′07.3″ E). Larvae were fixed in Carnoy’s solution (96% ethanol/glacial acetic acid, 3:1) and stored at −20 °C. Identification of the *An. messeae* species was carried out based on the length and the sequence of internal transcribed sequence 2 (ITS2) using the PCR-RFLP protocol [25]. Heads of larvae, previously soaked in 96% ethanol (at least for an hour), were used as a source of the genomic DNA template in a PCR reaction for species identification. To prevent inhibition of the PCR reaction by ethanol, each head was individually dried. Instead of extracting DNA from the heads, entire heads were used, with one head per tube. Afterward, the dried heads were placed in PCR mixtures. Salivary glands of 4th-instar *Anopheles* larvae were isolated in Carnoy’s solution, kept in a drop of 45% acetic acid for 10 min, covered by a coverslip with filter paper, and moderately squashed, as previously described [25]. For mapping of fixed inversions, the X1 karyotype of males and X11 karyotype of females of *An. messeae* were used. The obtained chromosome squashes were compared with the *An. messeae* standard chromosome map to ensure that they had the X1 arrangement (Figure S1) [25].

### 2.2. DNA Markers Development and Synthesis

The AatrE3 genome assembly of *An. atroparvus* [23,24] was used in VectorBase, which is part of VeuPathDB [26]. Nucleotide sequences of the *An. atroparvus* gene exons were chosen for developing primers for gene markers (Table S1). Primer pairs were developed using the online tool Primer-BLAST [27], with an emphasis on choosing primers with the best possible GC content and annealing temperatures between 52 °C and 56 °C. The melting temperatures of the two primers varied from 0.5 °C to 1 °C. Additionally, sequences with predicted PCR products ranging from 0.5 to 1 kilobase pairs were selected. The BLAST tool in VectorBase was used to confirm the uniqueness of the sequence and the absence of off-target sequences in the *An. atroparvus* AatrE3 genome. Validated primers were used for mapping to ensure the specificity of the probes. Using PCR kits (Biolabmix, Novosibirsk, Russia), the exons were amplified with the designed primers. Genomic DNA isolated from *An. atroparvus* was used as a template. With a 30 s annealing period, amplification was accomplished in 28 cycles. Gel electrophoresis was used to test amplified fragments for conformity to the predicted lengths. The PCR amplicons were purified to be used for preparing DNA probes.

### 2.3. DNA Probe Labeling, Fluorescence In Situ Hybridization (FISH), and Physical Mapping

Probe preparation and FISH were conducted as described previously [28,29]. Briefly, gene-specific DNA probes were labeled in the reaction with the Klenow fragment in the presence of heptamer primers with random nucleotide sequences and one of the modified nucleotides, TAMRA-5-dUTP or Biotin-11-dUTP (Biosan, Novosibirsk, Russia) [30]. To determine the order of the two selected genes and ensure the gap between them, we used two dyes simultaneously for the final visualization. The obtained DNA probes were precipitated in ethanol, dried, and dissolved in a hybridization mix (2× SSC, 10% SDS, 50% deionized formamide, 1% Tween 20). After overnight hybridization with a labeled DNA probe, chromosomal preparations underwent washing in the Block buffer (3% BSA, 2× SSC, 0.01% Tween 20) at 37 °C for 15 min, and the probes labeled with Biotin-11-dUTP were detected with Avidin-FITC (Sigma Aldrich, St. Louis, MO, USA) diluted 1:300 in the Block buffer for 1.5–2 h. After the final washing step in 2× SSC, 0.01% Tween 20, the slides were dried for 30 s and the antifade solution with DAPI (Abcam, Cambridge, MA, USA) was added under the coverslips. Chromosome preparations were analyzed with an Axio Imager Z1 fluorescence microscope (Carl Zeiss, Aalen, Germany). Digital microphotographs of chromosome spreads with fluorescent probe signals were captured with an AxioCam MRm CCD camera and AxioVision 4.7.1 software (Carl Zeiss, Germany). Physical mapping of *An. atroparvus* orthologs on polytene chromosomes of *An. messeae* was conducted using the standard cytogenetic map for *An. messeae* with the X1 arrangement [25].

### 2.4. Pair-Wise Analysis of Rearrangements and Identification of the Ancestral Arrangements

The Genome Rearrangements In Man and Mouse (GRIMM) program was used to calculate inversion distances in a pair-wise analysis between *An. atroparvus* and *An. messeae* [31]. The GRIMM (v. 2.01) software uses the Hannenhalli and Pevzner algorithms to compute the minimum number of rearrangement events and to find optimal scenarios for transforming one genome into another. The signed option for SB orientation was used. We define an SB as a region that contains no less than two genes in the same order independent of their orientation. Genome assemblies of multiple mosquito species from the VectorBase database were used for the ancestry analysis of chromosomal arrangements. Linear orders of orthologous sequences of 12 *Anopheles* species as well as *Culex quinquefasciatus* and *Aedes aegypti* were used as outgroups. Using the phylogenetic tree as a guide [9,23,32], the gene orders across BRs in the pair of the ingroup species *An. atroparvus* and *An. messeae* were compared with the gene orders in the outgroup species using a Genome Browser (Jbrowse by VectorBase). If the gene order in the BR was the same as in outgroups, then this gene order was considered ancestral.

### 2.5. Gene Ontology (GO) and Gene Enrichment Analysis

For GO analysis, we used a list of 181 genes of *An. atroparvus* (Table S2) and uploaded their IDs to the VectorBase strategy. Inside the strategy, we used the built-in tool “Gene Ontology Enrichment”. Genes were taken from SB2 (42 genes) and SB4 (139 genes). We covered all three domains of ontology: biological process, molecular function, and cellular component. Enrichment analysis was calculated based on hypergeometric distribution, supported by the Benjamini–Hochberg false discovery rate (FDR) and Bonferroni correction and represented by the enrichment table and relevant barplots. Also, we used *p*-value cutoff = 0.05 to only obtain enriched results.

### 2.6. Transposable Element (TE) and Simple Repeat Annotation

Using the Extensive de novo TE Annotator (EDTA) pipeline [33], a custom library of TEs was developed. Additionally, the RepeatModeler “http://www.repeatmasker.org/RepeatModeler/ (accessed on 21 November 2022)” was run to make the library complete. This library was used to annotate TEs and simple tandem repeats in the *An. atroparvus* AatrE3 assembly using RepeatMasker (v. 4.1.2) [34]. The rmsk2bed tool from BEDOPS (v. 2.4.41) was used to convert RepeatMasker .out files to convenient .bed files [35]. The resulting .bed files contained information about the beginning and the end of each repetitive DNA element and information about its type. For further analysis, all types of repetitive DNA were divided according to the RepeatMasker annotation into four groups: (1) TEs class I: retrotransposons; (2) TEs class II: DNA transposons; (3) unknown TEs; (4) simple repeats (Table 1).

The number of repetitive DNA elements from various groups was counted in each BR. Depending on the categories into which they were divided, repetitive DNA elements in each BR were visualized. In 50 kb genomic regions immediately upstream and downstream of each BR in the *An. atroparvus* genome, including the BR itself, bar plots were made to show how many repetitive DNA elements of each group were present. Each visualization bar was chosen to be equal in size to its corresponding BR. Genes were taken from the .gff3 AatrE3 annotation file retrieved from VectorBase, and the number of genes in each BR was displayed as described above [26].

### 2.7. Statistical Analyses

A statistical analysis was conducted to determine whether the difference in the density of repetitive DNA elements and genes in BRs was significant when compared to other regions of the X chromosome. The Poisson distribution was chosen to explain our data because the density of repetitive DNA elements or genes is a discrete value. The length of each BR was measured (ranging from 7 kb to 12 kb), and this length served as the chromosome’s overall bin size for the calculation of λ (the mean density of the repetitive DNA elements). Based on the obtained λ and x (the density of repetitive DNA elements in a single BR) for each BR, the value of P(x) was calculated. Moreover, coding regions were removed based on the coordinates obtained from the annotation .gff file of the AatrE3 genome assembly. Subsequently, the densities of repetitive DNA elements were recalculated using λ as the mean density in non-coding regions and x as the density in a single BR without coding regions. The resulting value of P(x) was utilized to assess the statistical significance of the difference in repetitive DNA density between BRs and non-coding regions on the X chromosome. The density of genes in each SB with a bin size of 100 kb (optimal for gene density calculation with appropriate detailing) was also estimated, and a one-way ANOVA test was run to determine if the density of genes in SBs varied significantly. For the statistical analysis, the scipy package (v. 1.10) of the Python programming language was used.

## 3. Results

### 3.1. Physical Mapping of Inversion Breakpoint Regions

**Figure 1:**
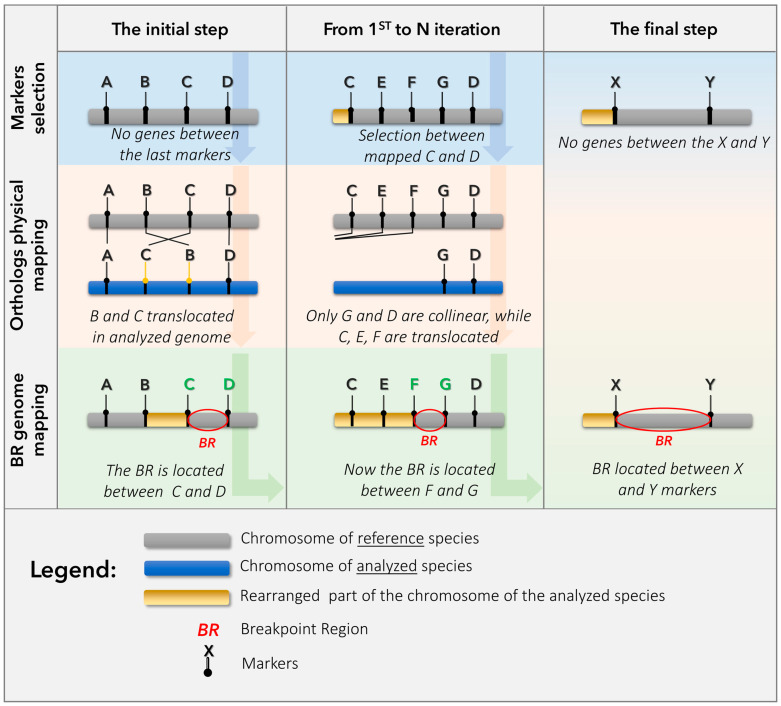
Scheme of iterative mapping of fixed inversions between *An. atroparvus* and *An. messeae*.

We designed and used an iterative physical mapping approach that sequentially localized marker genes of *An. atroparvus* on the polytene X chromosome of *An. messeae* with the purpose of identifying and narrowing down the inversion breakpoint areas (Figure 1).

**Figure 2:**
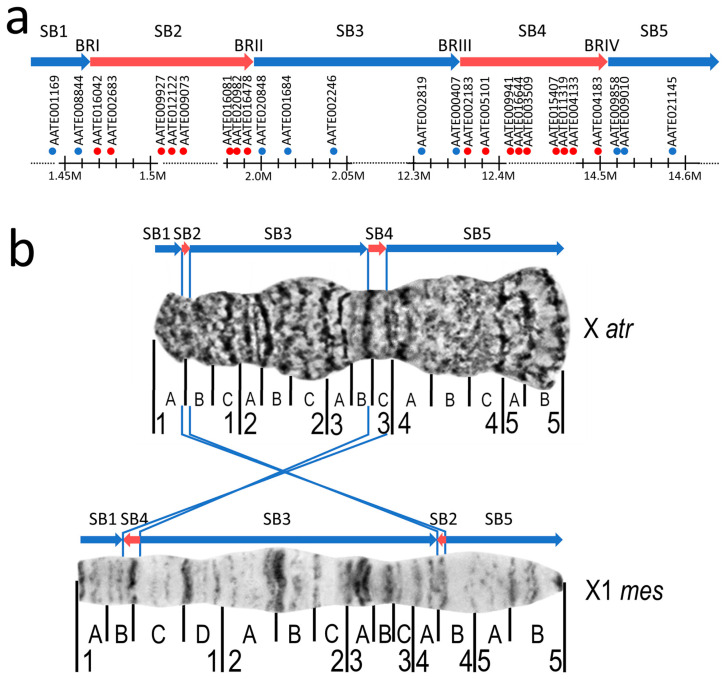
The physical map of gene markers, synteny blocks (SBs), and breakpoint regions (BRs) of the X chromosome inversions fixed between *An. atroparvus* and *An. messeae*. (**a**) Genomic position of genes, BRs and SBs in the *An. atroparvus* X chromosome. (**b**) Chromosomal positions of BRs and SBs in the *An. atroparvus* (**top**) and *An. messeae* (**bottom**) X chromosomes. Blue and red arrows represent collinear and inverted regions, respectively. Blue and red dots indicate genes located in collinear and inverted regions, respectively. The images of X chromosome maps of *An. atroparvus* (X atr) and *An. messeae* (X1 mes) are adapted from [25,36], respectively.

Iterative physical mapping was conducted in multiple steps. In the first step, we selected 17 gene markers spaced by about 1 Mb from each other [8]. These *An. atroparvus* markers were mapped to the X chromosome of *An. messeae* to identify the extent of gene-order collinearity between the species at the large scale. With exception of the three markers (AATE17741, AATE005236, and AATE010434), the gene orders were collinear between *An. Messeae* and *An. atroparvus,* indicating that these three genes are involved in fixed-chromosome rearrangements. In the consecutive steps, a series of FISH experiments were performed involving additional 36 gene markers, which allowed us to eventually map BRs more precisely. The selection of the new markers for each new FISH experiment was conducted based on the previous FISH results. For each FISH, three to four gene markers located within putative BRs were mapped to the *An. messeae* X chromosome to determine if they remained to be collinear or if they were translocated into new localizations in comparison with the *An. atroparvus* X chromosome. The mapping process was repeated to identify new gene markers between collinear and translocated segments of the genome. The range of the BRs became shorter after each round of hybridization until no mappable markers were identified within the BRs. Thus, the locations of a total of 53 genes in the X chromosome of *An. messeae* were determined by FISH (Table S1). This iterative mapping approach allowed us to identify five syntenic blocks (SB1-5) separated by four BRs (BR I-IV) (Figure 2a) of two large, nested inversions that are fixed between *An. atroparvus* and *An. messeae* (Figure 2b).

**Figure 3:**
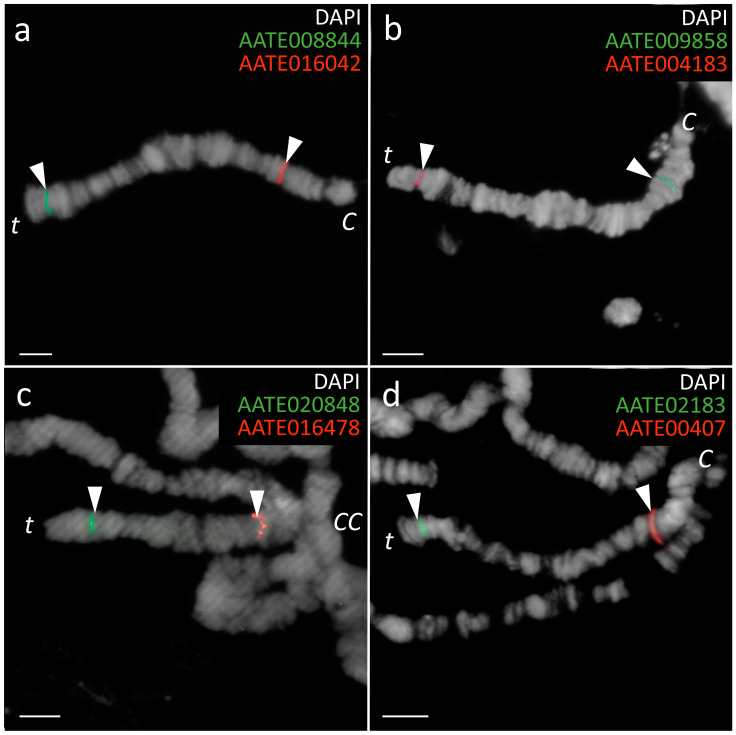
FISH of DNA probes developed based on the *An. atroparvus* genes that flank BRs of fixed rearrangements in the *An. messeae* X chromosome. (**a**) Markers for BRI. (**b**) Markers for BRIV. (**c**) Markers for BRII. (**d**) Markers for BRIII. The positions of the marker genes on the *An. messeae* X chromosomes are indicated by arrows. C—the centromere end of the X chromosome, t—the telomere end, CC—chromocenter. Scale bars—10 μm.

The final step of our FISH mapping of the *An. messeae* X chromosome identified genes that flank four BRs in the *An. atroparvus* AatrE3 genome assembly (Table S3). These gene pairs were adjacent in the *An. atroparvus* genome but were located in distant chromosomal regions in *An. messeae* (Figure 3). For example, AATE008844 of SB1 localized closely with AATE004183 of SB4 on the *An. messeae* X chromosome, indicating that these genes were moved by an inversion in *An. messeae*. Similarly, AATE016042 of SB2 localized closely with AATE009858 of SB5 on the *An. messeae* X chromosome (Figure 3). This rearrangement represents an outer inversion. AATE016478 of SB2 localized closely with AATE000407 of SB3 and AATE020848 of SB3 localized closely with AATE002183 of SB4 on the *An. messeae* X chromosome. This rearrangement represents an inner inversion nested within the larger outer inversion (Figure 2b).

### 3.2. Characterization of X Chromosome Rearrangements and Synteny Blocks

The four BRs identified on the X chromosome of *An. atroparvus* delineated five synteny blocks (SB1-5) of different sizes (Table 2). The SBs had no significant differences in gene densities; the *p*-value of ANOVA was 0.65, and the F-value was 0.61. The mean gene density in the X chromosome was 7.31 ± 3.68 per 100 kb with a maximum value of 20 genes per 100 kb.

SB1, SB3, and SB5 had the same order and orientation in *An. atroparvus* and *An. messeae*, whereas SB2 and SB4 differed in order and orientation between the two species (Figure 2b). To confirm that these five SBs resulted from two nested inversions, we used the GRIMM (v. 2.01) software [31] for the reconstruction of the rearrangement events that could have caused the observed differences. The following orders and orientations of SBs were used as an input for the GRIMM analysis:

>An. atroparvus X
1 2 3 4 5
>An. messeae X1
1 −4 3 −2 5

**Figure 4:**
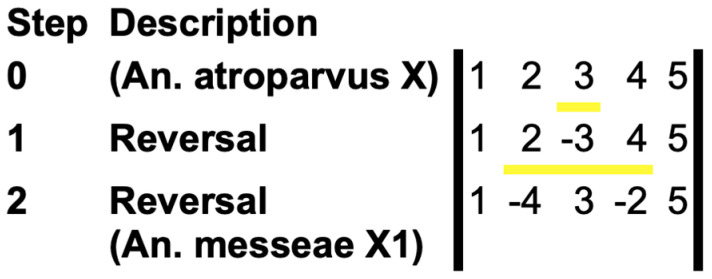
The GRIMM reconstruction of the X chromosome fixed inversions between *An. atroparvus* and *An. messeae*. The order and orientation of SBs are indicated by numbers and signs, respectively. The yellow lines show inversions (reversals).

The program identified two inversion events that transformed the putative standard X chromosome arrangement of *An. atroparvus* into the rearranged X chromosome of *An. messeae* (Figure 4).

The reconstructed inversions differed in length, and they used different pairs of BRs. The larger inversion (~13 Mb) involved SB2, SB3, and SB4 and used BRI and BRIV of *An. atroparvus*, whereas the smaller inversion (~10 Mb) involved only SB3 and used BRII and BRIII of *An. atroparvus*. Thus, the smaller inversion was nested inside the larger inversion. There are two possible scenarios of the rearrangement events. Either the small inversion occurred first, followed by the large inversion (as in Figure 4), or they occurred in the reverse order. It was not possible to reconstruct the actual order of events. The result of these rearrangements was a “swapping” of the SB2 and SB4 positions and a change in their orientation. These two SBs made up 16% of the euchromatic part of the X chromosome and only 10% of the entire X chromosome of *An. atroparvus*. At least 181 annotated genes residing in the SB2 and SB4 changed their position and orientation between the X chromosomes of *An. atroparvus* and *An. messeae*. Therefore, we decided to investigate whether SB2 and SB4 are enriched in genes of any particular biological roles or molecular functions.

**Figure 5:**
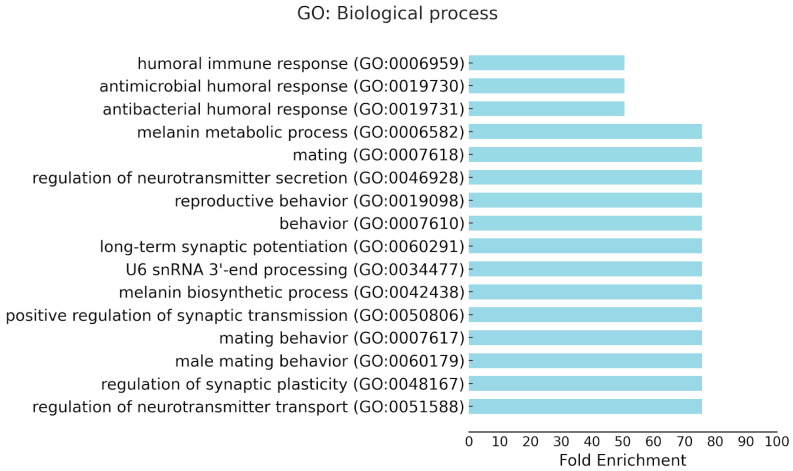
Enrichments of GO terms associated with biological processes for genes located in SB2 and SB4 of the *An. atroparvus* X chromosome. GO terms with fold enrichment more than 50 are shown.

Since the SB2 and SB4 changed their position and orientation between the X chromosomes of *An. atroparvus* and *An. messeae*, we analyzed GO term enrichments of their genes to understand the possible functional significance of the rearrangements. The results for the enriched biological process terms are shown in Figure 5 and Table S4. A significant *p*-value from Fisher’s exact test (*p* < 0.05) was found for 58 of the 345 GO terms. A significant adjusted *p*-value (Benjamini–Hochberg FDR < 0.05) was found for 13 of these terms including “antibacterial humoral response”, “antimicrobial humoral response”, “humoral immune response”, “immune response, “immune system process”, “defense response to other organism”, “response to other organism”, “response to external biotic stimulus, “biological process involved in interspecies interaction between organisms”, “response to biotic stimulus”, defense response”, defense response to bacterium”, and “response to bacterium”. However, the adjusted Bonferroni *p*-value for all 58 GO terms was not significant (Padj > 0.05). Therefore, caution should be exercised while interpreting these results, while they might still suggest biological relevance given that the enrichment is strong and biologically plausible. The fold enrichment and odds ratio quantify how much more likely it is to observe a particular GO term in the gene list compared to what would be expected by chance. A large fold enrichment (>50) and odds ratio (>151) was shown for the GO terms “antibacterial humoral response” and “humoral immune response”, indicating a strong association between the gene list and the GO term. Similarly, a large fold enrichment (>75) and odds ratio (infinity) was shown for the GO terms “regulation of neurotransmitter transport”, “regulation of synaptic plasticity”, “male mating behavior”, and “mating behavior”, indicating a strong association between these genes and the GO terms. We observed that genes in both SBs were enriched for identical GO terms, further suggesting the biological relevance of genes located in the inversions. For instance, the AATE021908 (SB2) and AATE021906 (SB4) genes were both associated with the GO terms “antibacterial humoral response”, “antimicrobial humoral response”, and “humoral immune response”.

The results of the analysis of the molecular function GO terms are shown in Table S5. Of the 189 identified GO terms, 29 GO terms had significant *p*-values from Fisher’s exact test (*p* < 0.05). However, adjusted *p*-values were not significant. Nevertheless, this analysis provided information on possible molecular functions of genes that we found among those associated with the enriched biological process terms. For example, AATE019391, involved in “immune response” and “defense response”, has the molecular function “peptidoglycan binding”. Also, genes AATE017047 and AATE021788, involved in the “ionotropic glutamate receptor signaling pathway”, have the molecular function “ionotropic glutamate receptor activity”.

Of the 73 identified GO terms associated with cellular components, 19 GO terms had significant *p*-values from Fisher’s exact test (*p* < 0.05) (Table S6). As expected, the AATE011784 gene, involved in “TOR signaling”, had the cellular components “TORC1 complex” and “TORC2 complex”. The AATE005672 gene, involved in “positive regulation of cell cycle process”, had the cellular components “supramolecular polymer/fiber” and “supramolecular complex”.

### 3.3. Identification of the Ancestral X Chromosome Arrangements

**Figure 6:**
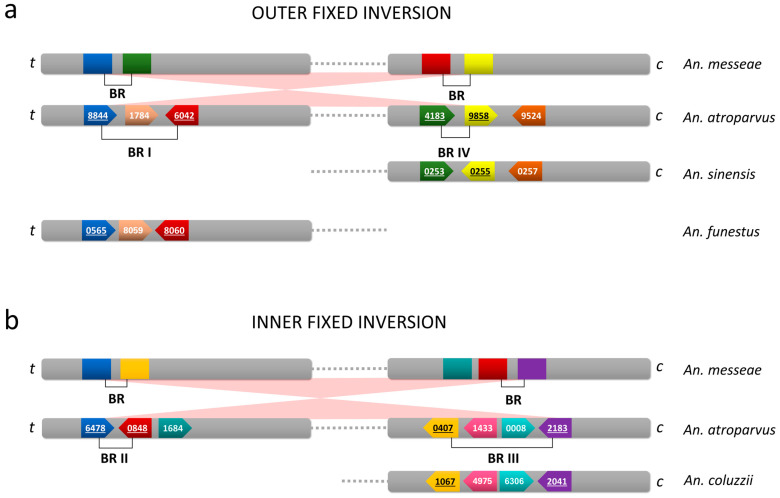
The scheme of gene order in breakpoint regions in the ingroup and outgroup species. (**a**) BRI and BRIV of the outer fixed inversion. (**b**) BRII and BRIII of the inner fixed inversion. The genes are depicted by colored blocks and identified by the last 4 digits of the IDs. The brackets indicate the BRs between the two flanking genes in *An. atroparvus*, and the underlined gene names are used to identify the BRs. The orientation of genes in *An. messeae* is unknown. ‘t’ represents telomere and ‘c’ represents centromere.

We used the phylogenetic tree for species of the *Anopheles* genus, for which genome assemblies are available [18,27,28], to visualize the evolution of chromosomal positions and linear orders of orthologs of genes flanking BRs in *An. atroparvus* and *An. messeae* (Figures S1–S4). According to the tree, *Culex* and *Aedes* are outgroups, and the American mosquitoes *An. albimanus* and *An. darlingi* represent the basal lineage of the *Anopheles* genus. The most closely related species to the Maculipennis subgroup is the Asian mosquito *An. sinensis*. To reconstruct the ancestral X chromosome arrangements of the Maculipennis subgroup, we compared the gene orders in the BRs in ingroup species (*An. atroparvus* and *An. messeae*) with the gene orders in species outside the Maculipennis subgroup. If the gene order in the BR was the same as in outgroups, then this gene order was considered ancestral. The main factors affecting the preservation of gene orders in our analysis included evolutionary distance from the Maculipennis subgroup and the quality of genome assemblies. The analysis shows that outgroup species support the ancestry of gene orders found in *An. atroparvus* but not in *An. messeae* (Figure 6). This is demonstrated for BRI, BRIII, and BRIV. Our analysis of gene orders in BRII could not provide support for the ancestry of either *An. atroparvus* or *An. messeae*. The gene order in BRIII is the most evolutionarily conserved among the BRs (Figure S4). However, we were unable to map AATE001433 and AATE020008 of BRIII on the chromosomes of *An. messeae* using FISH.

### 3.4. The Genomic Content in the Neighborhoods of the Breakpoint Regions

**Figure 7:**
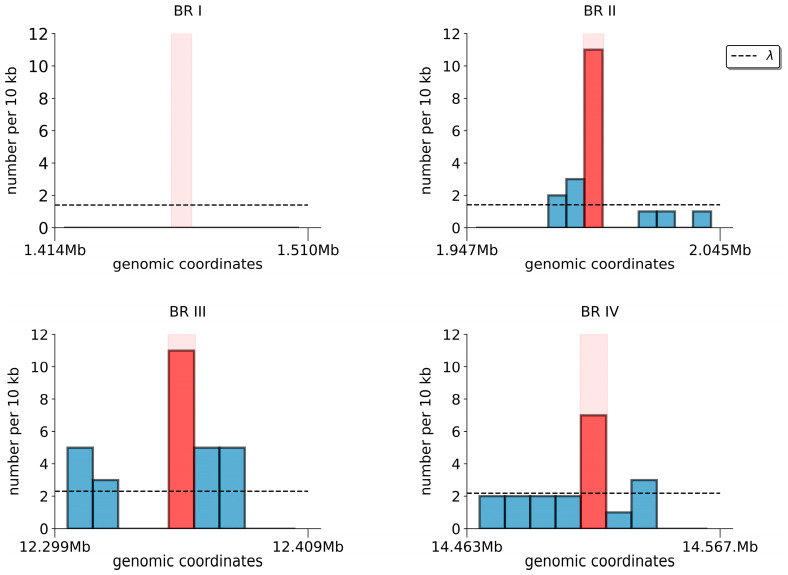
Density of DNA transposons (Class II TEs) in the BRs and approximately 100 kb genomic neighborhoods of the BRs in the X chromosome of *An. atroparvus*. Light pink bars show the position of BRs within genomic neighborhoods. Red and blue bars show the density of TEs in BRs and genomic neighborhoods, respectively. Genomic neighborhoods are defined as 50 kb genomic regions located immediately upstream and downstream of each BR. λ is a mean density of DNA transposons over the entire chromosome with bin size equal to the corresponding BR size.

Since the genome sequence of *An. messeae* is not yet available, we analyzed the genomic content in the neighborhoods of the BRs using the genome sequence of *An. atroparvus*. These neighborhoods were defined as 50 kb genomic regions located immediately upstream and downstream of each BR. We analyzed these neighborhoods for the presence of TEs and tandem repeats, which are known to cause chromosomal rearrangements [37,38,39,40]. Most TEs in dipteran insects belong to two major classes that are transposed by using an RNA transposition intermediate (Class I) and the “cut and paste” mechanism (Class II) [41]. The density of the class I TEs was low in the X chromosome of *An. atroparvus,* and only a single retroelement was found near one of the BRs (BRI). However, TEs of Class II were more abundant in the X chromosome, and the density of DNA transposons was significantly higher in BRII, BRIII, and BRIV in comparison with the average density for the X chromosome with the following *p*-values: 2.7 × 10^−7^, 2.3 × 10^−5^, and 5.2 × 10^−3^, respectively (Figure 7).

BRI and BRIII were characterized by a high density of simple repeats in comparison with BRII and BRIV (Figure S5). All the BR regions had different TE content and simple repeat content (Table 3). BRI contained no TEs, but the other three BRs had at least one copy of CACTA TIR transposon DNA/DTC. Multiple copies of DNA PIF Harbinger MITE/DTH were found in BRII and BRIII, whereas BRIV had four Helitrons. Tandem repeats were found in every BP as one to six nucleotide repeats (Table 3). The density of genes was significantly higher only in BRIII in comparison with the average gene density for the X chromosome, with a *p*-value of 0.044 (Figure S6).

## 4. Discussion

A growing body of research indicates that chromosome rearrangements are significant factors in species evolution and adaptation [42,43,44]. Data obtained from various organisms suggest that chromosomal polymorphism is a mechanism that species use to adapt rapidly to climate changes [45,46]. Cytogenetic studies on malaria mosquitoes have shown non-uniform inversion distribution among species and chromosomal arms [47,48,49,50]. Evolutionary genomic analyses in *Anopheles* species have shown that fixed inversions accumulate about three times faster on the X chromosome than on autosomes [23,50,51]. It has been demonstrated that the fixation rate of underdominant and advantageous partially or fully recessive rearrangements should be higher for the X chromosome (due to the hemizygosity of males) than for the autosomes [52]. This study utilized the iterative physical mapping approach to map BRs of fixed X chromosomal inversions between *An. atroparvus* and *An. messeae* (Figure 1). DNA probes were designed based on *An. atroparvus* gene sequences and mapped to *An. messeae* polytene chromosomes using FISH. The density of gene markers was increased with each iteration mapping step to precisely identify BRs of fixed rearrangements between a species with a reference genome and a target species. This approach considers only multiple linked markers to minimize the misidentification of BRs caused by single-gene transpositions.

Two nested inversions were identified in the X chromosome, which were fixed in the *An. messeae* lineage after its separation from other species of the Maculipennis subgroup about 7.9 million years ago [8]. The comparison of gene orders in the BRs with those in outgroup species has shown that *An. atroparvus* has ancestral arrangements and *An. messeae* has derived arrangements (Figure 6). These two inversions are specific to *An. messeae* as they have not been identified in its sister species, *An. maculipennis*. No autosomal inversions were fixed in the *An. messeae* lineage after its separation from *An. maculipennis* and *An. melanoon*. Only one autosomal inversion became fixed after the split between the European clade and the Northern Eurasian clade about 10.5 million years ago [8]. According to our reconstruction, the X chromosome of *An. messeae* underwent two simultaneous or consecutive inversion events, but the order of these events could not be determined without analysis of the BRs in the *An. messeae* genome. The X1 arrangement is fixed in *An. messeae* species, whereas the X1 arrangement is polymorphic in the sister species, *An. daciae* [17,26]. Our previous high-resolution cytogenetic mapping of polytene chromosomes with the X0 and X1 arrangements in *An. daciae* demonstrated that the BRs of the polymorphic inversion X1 do not coincide with the BRs of the fixed inversions in *An. messeae* [26]. Therefore, the X0 arrangement originated independently in *An. daciae* based on the X1 arrangement shared by both species.

Inversions are generated by two major mechanisms, ectopic recombination and staggered breaks [22,53,54]. The staggered break mechanism requires no specific sequence for realization. In contrast, the ectopic recombination mechanism occurs during recombination between two homologous opposite-oriented genomic sequences (often TEs) on the same chromosome. As there is no sequenced genome available for *An. messeae*, we analyzed BRs using the *An. atroparvus* genome. The enrichment of TEs and other repetitive sequences in BRs in *An. atroparvus* may suggest the presence of “hot spots” for rearrangements in the ancestral X chromosome. We found that BR neighborhoods contain a variety of TEs (Figure 7). Some BRs contain TEs belonging to the same families (Table 3), which could be a prerequisite to inversion origination by the ectopic recombination mechanism. The abundance of TEs within inversion BRs is typical for species of the *An. gambiae* complex and *An. stephensi* [55,56]. We found DNA transposons but no retrotransposons in the BRs of *An. atroparvus*. This is in contrast with *An. gambiae* in which LTR-Gypsy elements were identified in the 2La inversion BRs [57]. The analysis of gene density in the neighborhoods surrounding BRs indicates that BRs are located in gene-rich genomic regions. This finding is consistent with the recent observation of an enrichment of synteny breakpoints in regions with high gene density in the genomes of multiple *Anopheles* species [58].

The outer inversion captured 901 genes, which accounted for 75% of the X chromosome, while the inner inversion captured 722 genes, which accounted for 59% of the X chromosome, based on the *An. atroparvus* genome. This high percentage of the captured genes by the inversions in *Anopheles* is consistent with the larger sizes of X chromosomal inversions compared to autosomal inversions demonstrated in *Drosophila* [59,60]. Studies have demonstrated that intermediate-to-large size inversions are maintained as balanced polymorphisms via associative overdominance and contribute to the local adaptation of species [60]. Therefore, it is reasonable to assume that large X chromosomal inversions had an adaptive value for ancestral populations of *An. messeae* before fixation. Chromosomal inversions suppress genetic recombination in the rearranged region, reducing gene flow between inverted and non-inverted variants [1,61]. This results in different combinations of gene alleles, commonly known as “supergenes,” being linked to each arrangement [62].

Although the two nested X chromosome inversions were of considerable size, the gene order and orientation differed only in two small synteny blocks, SB2 and SB4 (Figure 2), between *An. atroparvus* and *An. messeae*. These two blocks contained 181 genes, which representing 10% of the X chromosome. Our analysis of GO term enrichments in SB2 and SB4 provides information about the possible functional significance of the X chromosomal rearrangements. The study revealed pathways associated with the regulation of the immune system and neurotransmitter receptor activities in malaria mosquitoes (Figure 5 and Table S4). The pathways related to immune system regulation could indicate a possible role of fixed X chromosomal inversions in the evolution of the protective response against malaria parasites. In Europe, *An. atroparvus* was almost always associated with endemic malaria, while *An. messeae* was reported as a vector of some importance when its density was high, especially in regions with a lot of livestock [63]. An experimental study has demonstrated that both *An. atroparvus* and *An. messeae* were highly susceptible to strains of *Plasmodium vivax* from Africa, Asia, and South America in the laboratory. The oocyst–sporozoite index (i.e., the percentage of mosquitoes with oocysts that then develop into sporozoites) in *An. messeae* (21 ± 7%) was significantly lower than that in *An. atroparvus* (62 ± 4%), indicating that oocysts develop into sporozoites much less frequently in *An. messeae* than in *An. atroparvus* [64]. This may be due to differences in immune response between the species. The significant enrichment of immune system-related genes in the inverted syntenic blocks could provide insights into understanding the molecular mechanisms behind inter-species variation in mosquito susceptibility to malaria infection.

Neurotransmitter receptor activities play a crucial role in regulating behaviors, including reproductive and male mating behavior. This suggests that genetic regulation directed from SB2 and SB4 are important for mosquito reproduction. Interestingly, *An. atroparvus* and *An. messeae* differ in male mating behavior. It has been documented that *An. atroparvus* does not require swarming prior to mating and mates almost exclusively indoors, whereas *An. messeae* mates only outdoors [63]. The X chromosome may also play a role in the evolution of mating behavior in other *Anopheles* species. A study of GO term enrichment in the X chromosome of *An. gambiae* identified genes involved in pre-mating isolation. These genes encode proteins with molecular and signal transduction activity, which are crucial components of olfaction that play a major role in mate recognition [50]. Thus, our GO analysis identified two promising directions for further research. This analysis narrowed down the list of potentially significant genes. In another study, X-linked genes encoding signal transduction proteins exhibited differential expression between virgin females of two incipient species of *An. gambiae* that differ in swarming behavior [65]. The rapid generation and fixation of inversions on the X chromosome may facilitate speciation in *Anopheles* by differentiating alleles inside of the inverted regions, as has been shown in *Drosophila* [66]. Compared to *An. atroparvus*, *An. messeae* has a much wider range of habitat, including the harsh climatic zones of western Siberia, characterized by short, cool summers and much shorter life cycles. It is possible that the fixed and polymorphic chromosomal inversions played a role in the adaptation of *An. messeae* mosquitoes to their environment.

## 5. Conclusions

Chromosomal inversions play an important role in genome evolution, speciation, and adaptation of organisms to diverse environments. Mapping and characterizing inversions can help describe rearrangement mechanisms and identify genes involved in species diversification. While cytogenetic characterization has identified autosomal inversions fixed among species within the Maculipennis subgroup based on distinct chromosomal banding patterns, elucidating the number and location of chromosomal rearrangements within the X chromosome has been difficult due to disparate banding patterns. In this study, we characterized chromosomal rearrangements that differentiate *An. atroparvus* and *An. messeae*. By employing an iterative mapping approach, we identified two nested X chromosome inversions that have been fixed in the *An. messeae* lineage. These inversions yielded five syntenic blocks, with only two small blocks, housing 181 annotated genes in the *An. atroparvus* genome, exhibiting an altered position and orientation within the X chromosome. We examined the functional implications of two small blocks, revealing potential roles in regulating the immune system and neurotransmitter receptor activities that are crucial for mating behavior. These findings offer insight into the underlying mechanisms of mosquito biology and behavior, with implications for disease transmission. This work highlights promising avenues for future research and the development of novel hypotheses by identifying specific genomic regions enriched in genes related to immune response and reproductive behavior. Our genomic analysis suggests a potential mechanism for the generation of inversions through ectopic recombination between transposable elements or other repetitive sequences located in breakpoint regions. This study underscores the efficacy of employing physical genome mapping techniques to discern species differentiation through chromosomal rearrangements in insects with polytene chromosomes.

## Figures and Tables

**Table 1:** Groups of TEs based on the summary file produced by EDTA.

Repetitive DNA Family	Repeat Name
TEs I: Retrotransposons	LTRs: Gypsy, Copia, Unknown; LINEs
TEs II: DNA TEs	TIRs: CACTA, Mutator, PIF_Harbinger, Tc1_Mariner, hAT; helitron
Unknown TEs	Repeat_region
Simple repeats	Low_complexity, Simple_repeat

**Table 2:** The genomic lengths, gene counts, and gene densities in the five synteny blocks in the X chromosome of the *An. atroparvus* AatrE3 genome.

SB	Length of SB, bp	Gene Counts in SB	Mean Gene Density in SB (per 100 kb)
1	1,458,895	122	8.36
2	526,550	42	7.97
3	10,348,225	722	6.98
4	2,148,753	137	6.38
5	3,273,690	199	6.6
Total	17,756,113	1222	7.26

**Table 3:** Repetitive DNA content and coverage in breakpoint regions according to the RepeatMasker annotation.

BR	Simple Repeats	Transposable Elements	Total Coverage of Repetitive DNA in BR
I	(CAG)_8_, (CAG)_10_, (AC)_25_, (GA)_11_, (CTC)_9_, (TTC)_13_,(CCAGC)_5_, (TATTTA)_8_, A-rich_93_, A-rich_39_, A-rich_42_, A-rich_36_	None	7.4%
II	(TTC)_12_	CACTA TIR transposon DNA/DTC (1), DNA hAT DNA/DTA (1), DNA PIF Harbinger MITE/DTH (8), Helitron (1), Unknown (1)	18.6%
III	(CGATGC)_9_, (GCCACC)_7_, (TGC)_30_, (TAT)_9_, (CT)_14_, (GGATT)_6_, I_27_	CACTA TIR transposon DNA/DTC (3), DNA PIF Harbinger MITE/DTH (2), hAT TIR transposon DNA MITE/DTA (2), hAT TIR transposon DNA/DTA (1), DNA/DTT (1), Helitron (2)	14.4%
IV	©_24_	CACTA TIR transposon DNA/DTC (2), Mutator TIR transposon (1), Helitron (4), Unknown (2)	23.9%

Note: BR—breakpoint region. The number in the brackets indicates the number of TEs.

## Data Availability

All the data are available in the manuscript and Supplementary Materials. The data and materials used in this study data are also archived in a publicly accessible repository at “https://doi.org/10.5281/zenodo.10940345 (accessed on 8 April 2024)”.

## Supplementary Materials

The following supporting information can be downloaded at https://www.mdpi.com/article/10.3390/insects15050312/s1. Figure S1: Genomic linear order of orthologs of genes flanking BRI in *An. atroparvus*; Figure S2: Genomic linear order of orthologs of genes flanking BRIV in *An. atroparvus*; Figure S3: Genomic linear order of orthologs of genes flanking BRII in *An. atroparvus*; Figure S4: Genomic linear order of orthologs of genes flanking BRIII in *An. atroparvus*; Figure S5: Density of simple repeats in the BRs and approximately 100 kb genomic neighborhoods of the BRs in the X chromosome of *An. atroparvus.* Light pink bars show the position of BRs within genomic neighborhoods. Red and blue bars show the density of simple repeats in BRs and genomic neighborhoods, respectively. Genomic neighborhoods are defined as 50 kb genomic regions located immediately upstream and downstream of each BR. λ is the mean density of simple repeats over the entire chromosome with bin size equal to corresponding BR size; Figure S6: Gene density in the BRs and approximately 100 kb genomic neighborhoods of the BRs in the X chromosome of *An. atroparvus*. Light pink bars show the position of BRs within genomic neighborhoods. Red and blue bars show the density of genes in BRs and genomic neighborhoods, respectively. Genomic neighborhoods are defined as 50 kb genomic regions located immediately upstream and downstream of each BR. Λ is a mean density of genes over the entire chromosome with bin size equal to corresponding BR size; Table S1: The *An. atroparvus* genes are used as markers for the physical mapping of breakpoint regions of fixed rearrangements in the X chromosome of *An. messeae*; Table S2: List of genes located in SB2 and SB4 of the *An. atroparvus* X chromosome; Table S3: The coordinates of the BR-flanking gene markers in the *An. atroparvus* genome assembly; Table S4: Enriched GO biological process terms in SB2 and SB4 of the *An. atroparvus* X chromosome; Table S5: Enriched GO molecular function terms in SB2 and SB4 of the *An. atroparvus* X chromosome; Table S6: Enriched GO cellular component terms in SB2 and SB4 of the *An. atroparvus* X chromosome.

## Abbreviations List

AatrE3	*Anopheles atroparvus* EBRO genome assembly
BR	Breakpoint region
FISH	Fluorescent in situ hybridization
GO	Gene ontology
ITS2	Internal transcribed spacer 2 (of the small-subunit ribosomal RNA)
RFLP	Restriction fragment length polymorphism
SB	Synthetic block
TE	Transposable elements
UTR	Untranslated region
