# Supplementary material for: Two Nested Inversions in the X Chromosome Differentiate the Dominant Malaria Vectors in Europe, Anopheles atroparvus and Anopheles messeae
Source: Insects. 2024 Apr 26;15(5):312. doi: 10.3390/insects15050312 (PMC11122324; doi:10.3390/insects15050312)
Supplement: Supplementary file 1 [file insects-15-00312-s001.zip › Supplementary figures with captures.pdf]

Supplementary figures

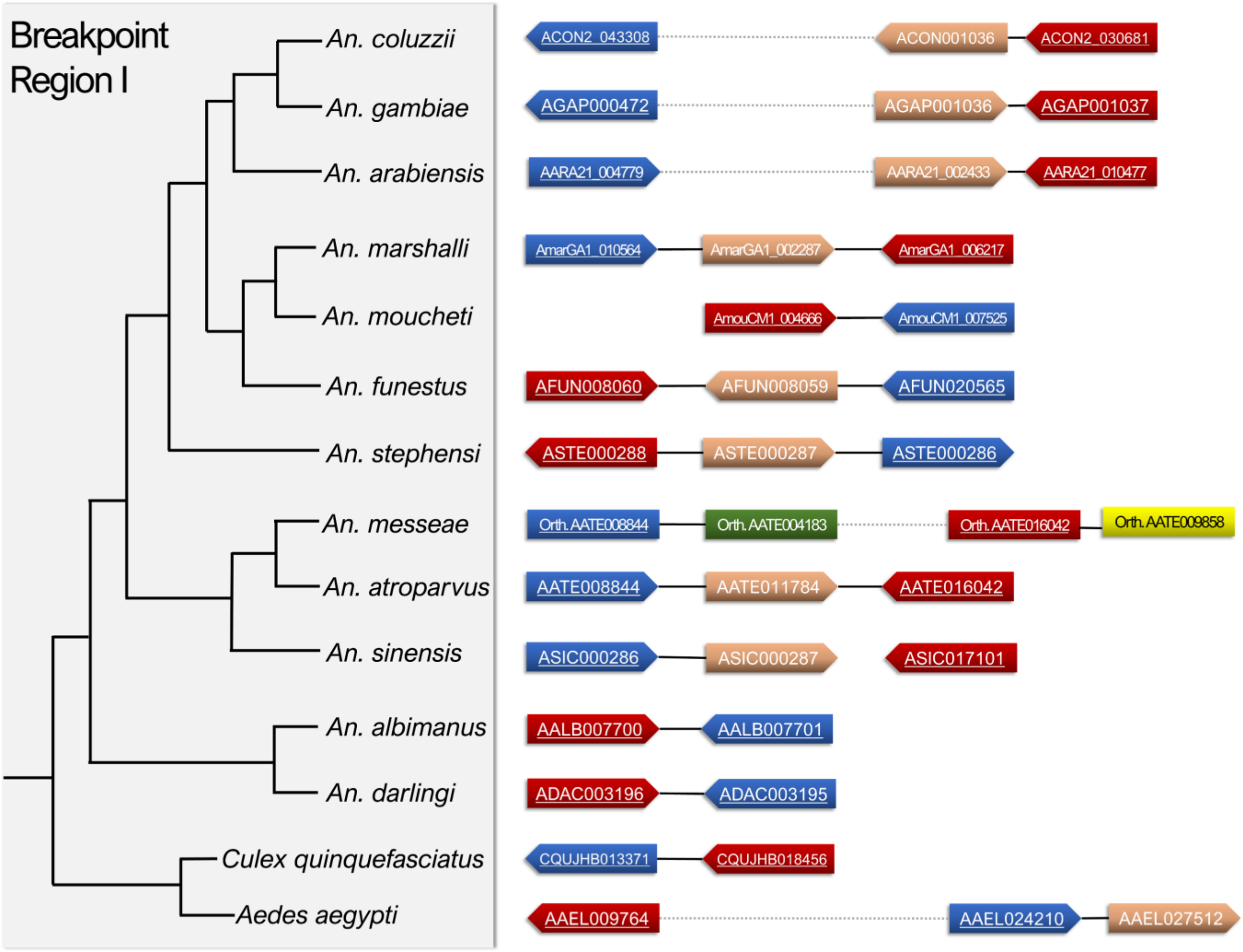

Figure S1. Genomic linear order of orthologs of genes flanking the BRI in An. atroparvus. Orthologous genes are denoted by similar colors. Gene IDs are shown as assigned by each genome assembly. Genes flanking inversion BRs are underlined. A solid black line indicates close proximity between genes, while a dotted line indicates a distance greater than 1 million base pairs. The gene's position on the plus strand is indicated by the right-pointing arrow, while the position on the minus strand is indicated by the left-pointing arrow.

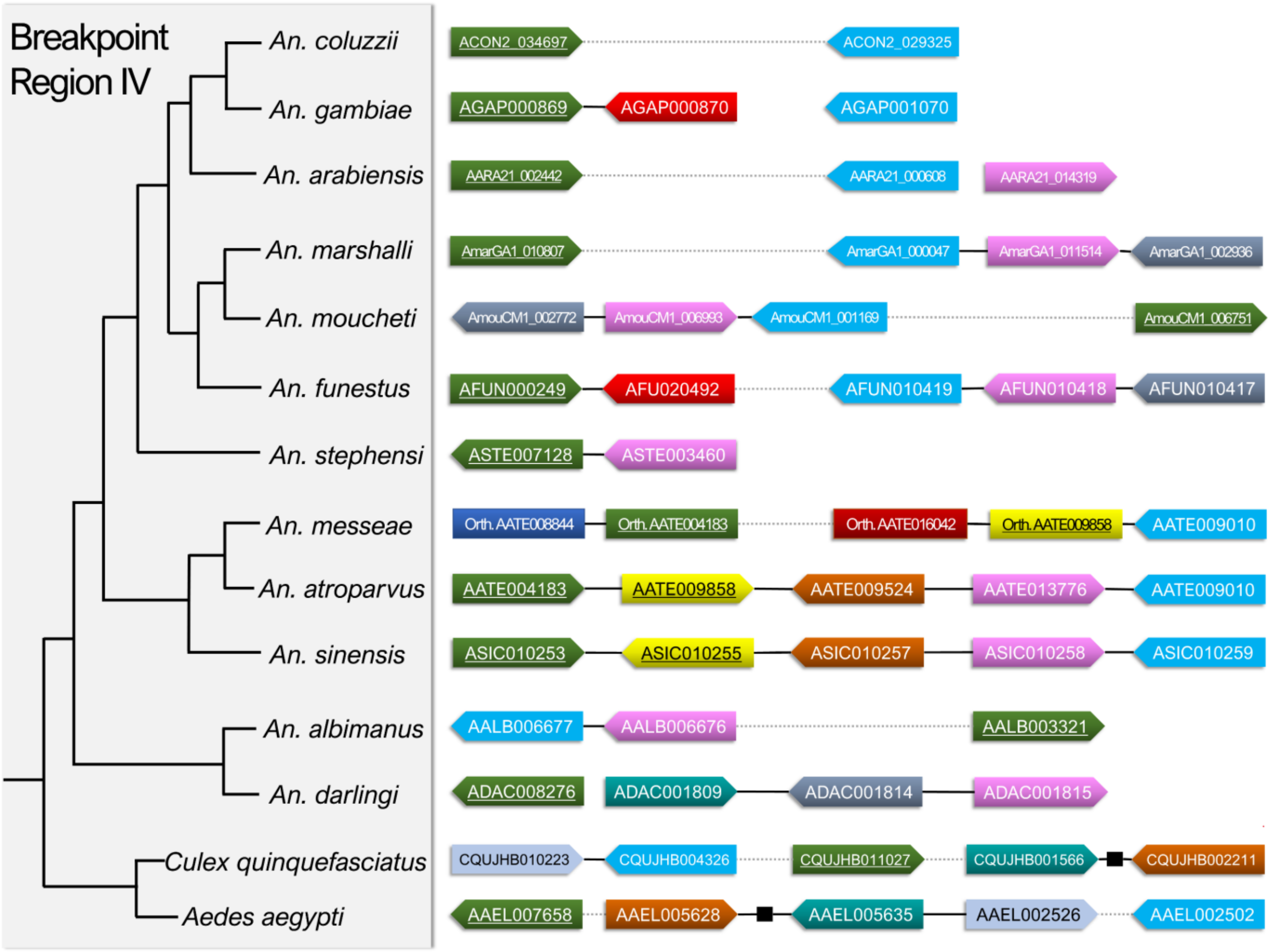

Figure S2. Genomic linear order of orthologs of genes flanking the BRIV in An. atroparvus. Orthologous genes are denoted by similar colors. Gene IDs are shown as assigned by each genome assembly. Genes flanking inversion breakpoint regions are underlined. A solid black line indicates close proximity between genes, while a dotted line indicates a distance greater than 1 million base pairs. The gene's position on the plus strand is indicated by the right-pointing arrow, while the position on the minus strand is indicated by the left-pointing arrow.

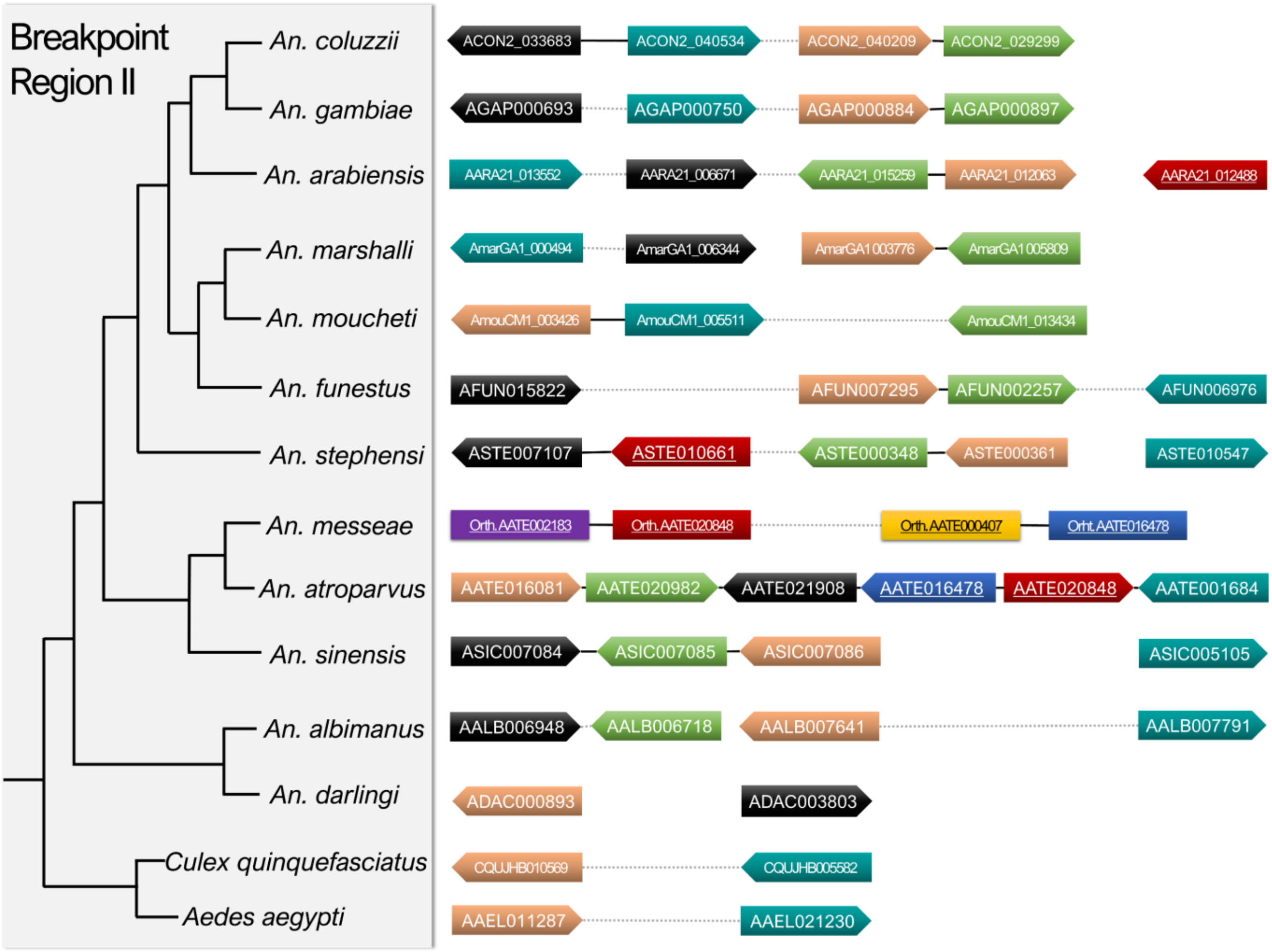

Figure S3. Genomic linear order of orthologs of genes flanking the BRII in An. atroparvus. Orthologous genes are denoted by similar colors. Gene IDs are shown as assigned by each genome assembly. Genes flanking inversion BRs are underlined. A solid black line indicates close proximity between genes, while a dotted line indicates a distance greater than 1 million base pairs. The gene's position on the plus strand is indicated by the right-pointing arrow, while the position on the minus strand is indicated by the left-pointing arrow.

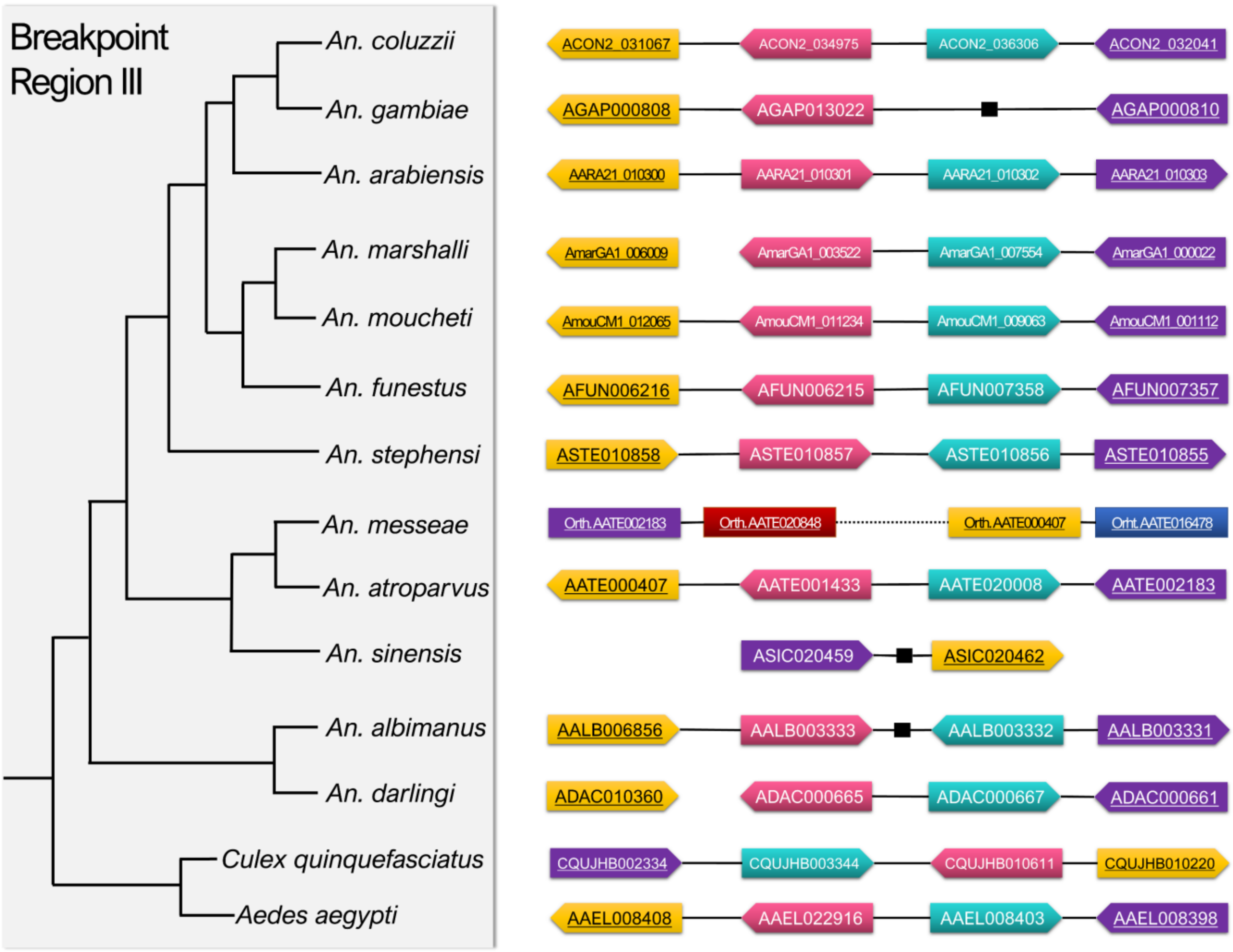

Figure S4. Genomic linear order of orthologs of genes flanking the BRIII in An. atroparvus. Orthologous genes are denoted by similar colors. Gene IDs are shown as assigned by each genome assembly. Genes flanking inversion BRs are underlined. A solid black line indicates close proximity between genes, while a dotted line indicates a distance greater than 1 million base pairs. The gene's position on the plus strand is indicated by the right-pointing arrow, while the position on the minus strand is indicated by the left-pointing arrow.

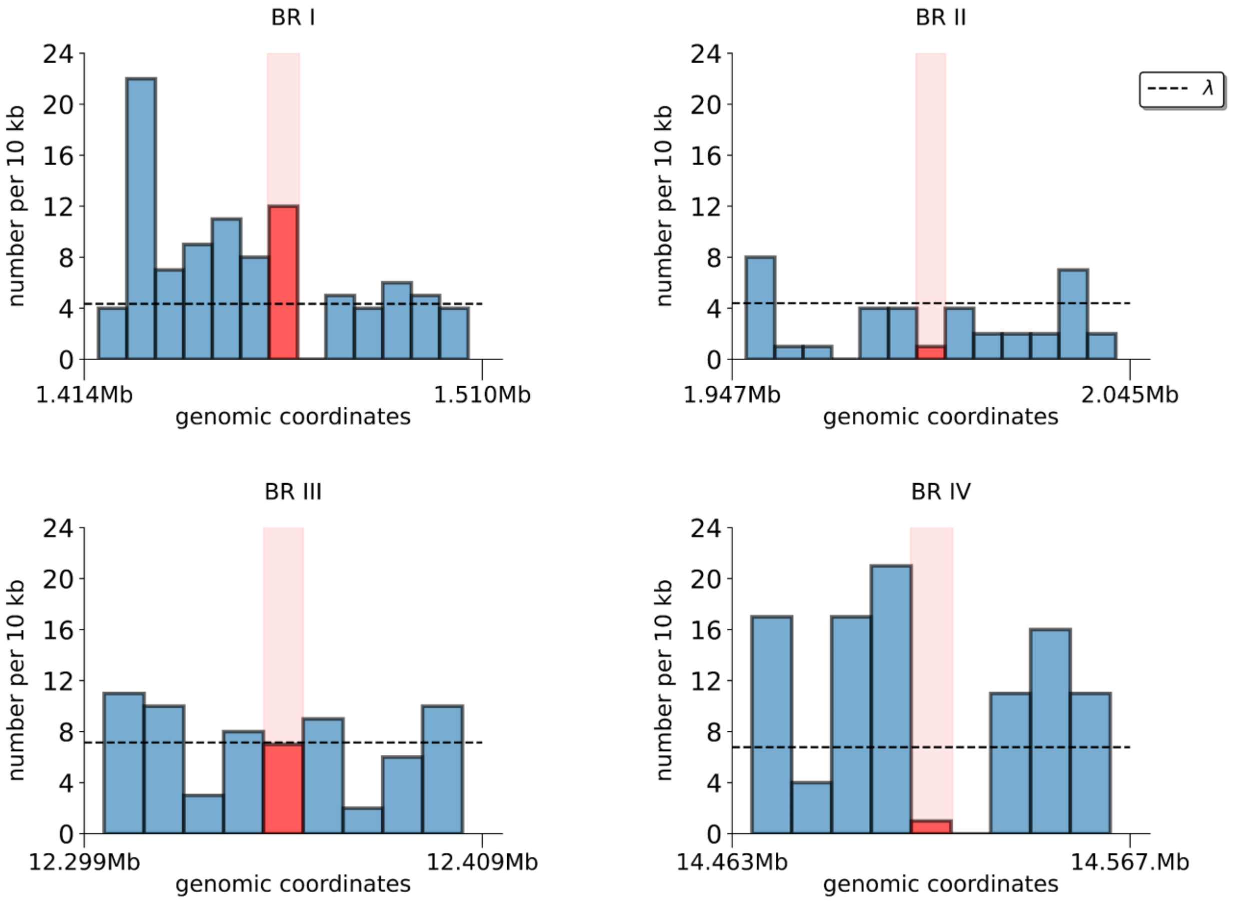

Figure S5. Density of simple repeats in the BRs and approximately 100-kb genomic neighborhoods of the BRs in the X chromosome of An. atroparvus. Light pink bars show the position of BRs within genomic neighborhoods. Red and blue bars show the density of simple repeats in BRs and genomic neighborhoods, respectively. Genomic neighborhoods are defined as 50-kb genomic regions located immediately upstream and downstream of each BR. λ is a mean density of simple repeats over the entire chromosome with bin size equal to corresponding BR size.

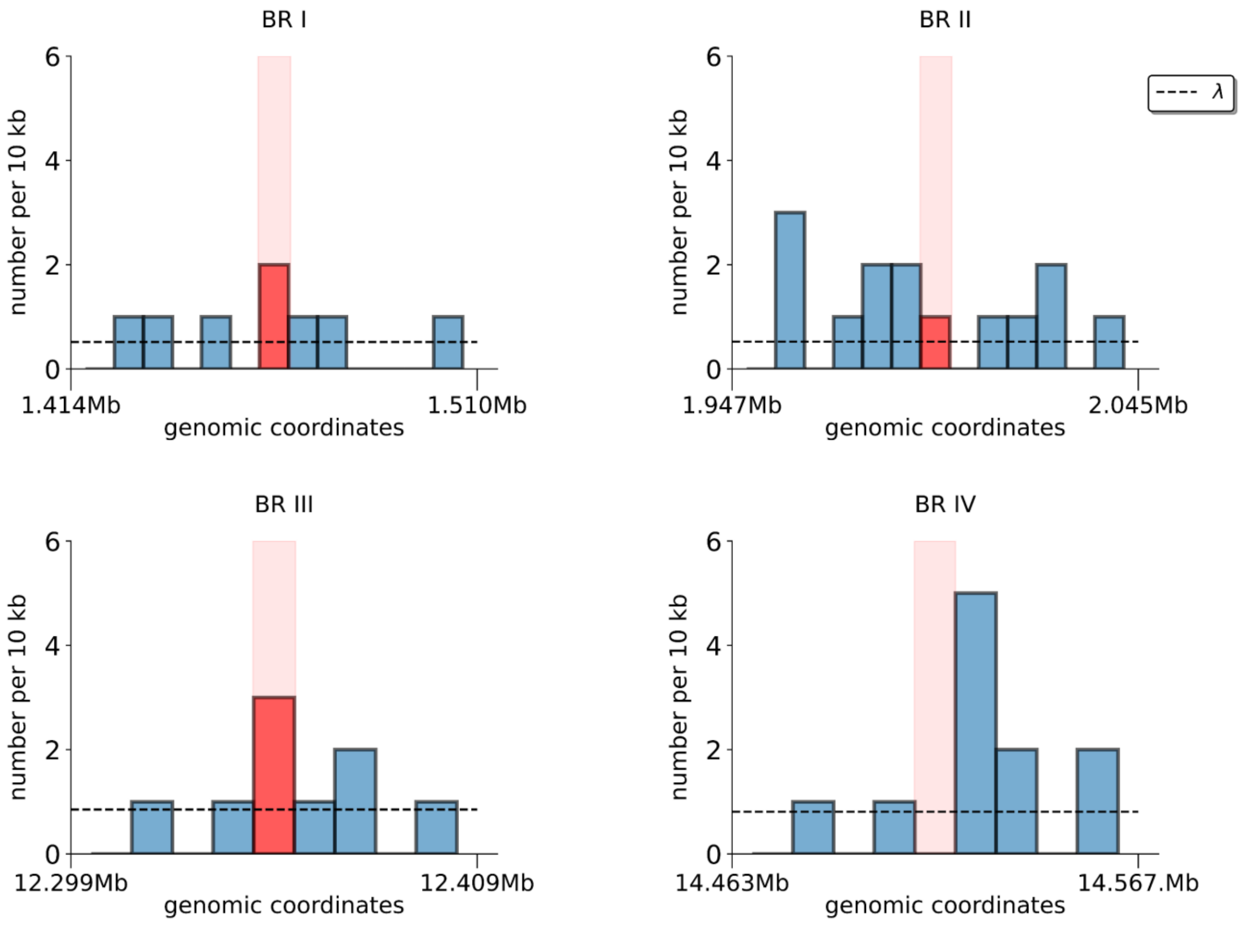

Figure S6. Gene density in the BRs and approximately 100-kb genomic neighborhoods of the BRs in the X chromosome of An. atroparvus. Light pink bars show the position of BRs within genomic neighborhoods. Red and blue bars show the gene density in BRs and genomic neighborhoods, respectively. Genomic neighborhoods are defined as 50-kb genomic regions located immediately upstream and downstream of each BR. λ is a mean density of genes over the entire chromosome with bin size equal to corresponding BR size.
