# Supplementary figures and images for: Two Nested Inversions in the X Chromosome Differentiate the Dominant Malaria Vectors in Europe, Anopheles atroparvus and Anopheles messeae
Source: Insects. 2024 Apr 26;15(5):312. doi: 10.3390/insects15050312 (PMC11122324; doi:10.3390/insects15050312)

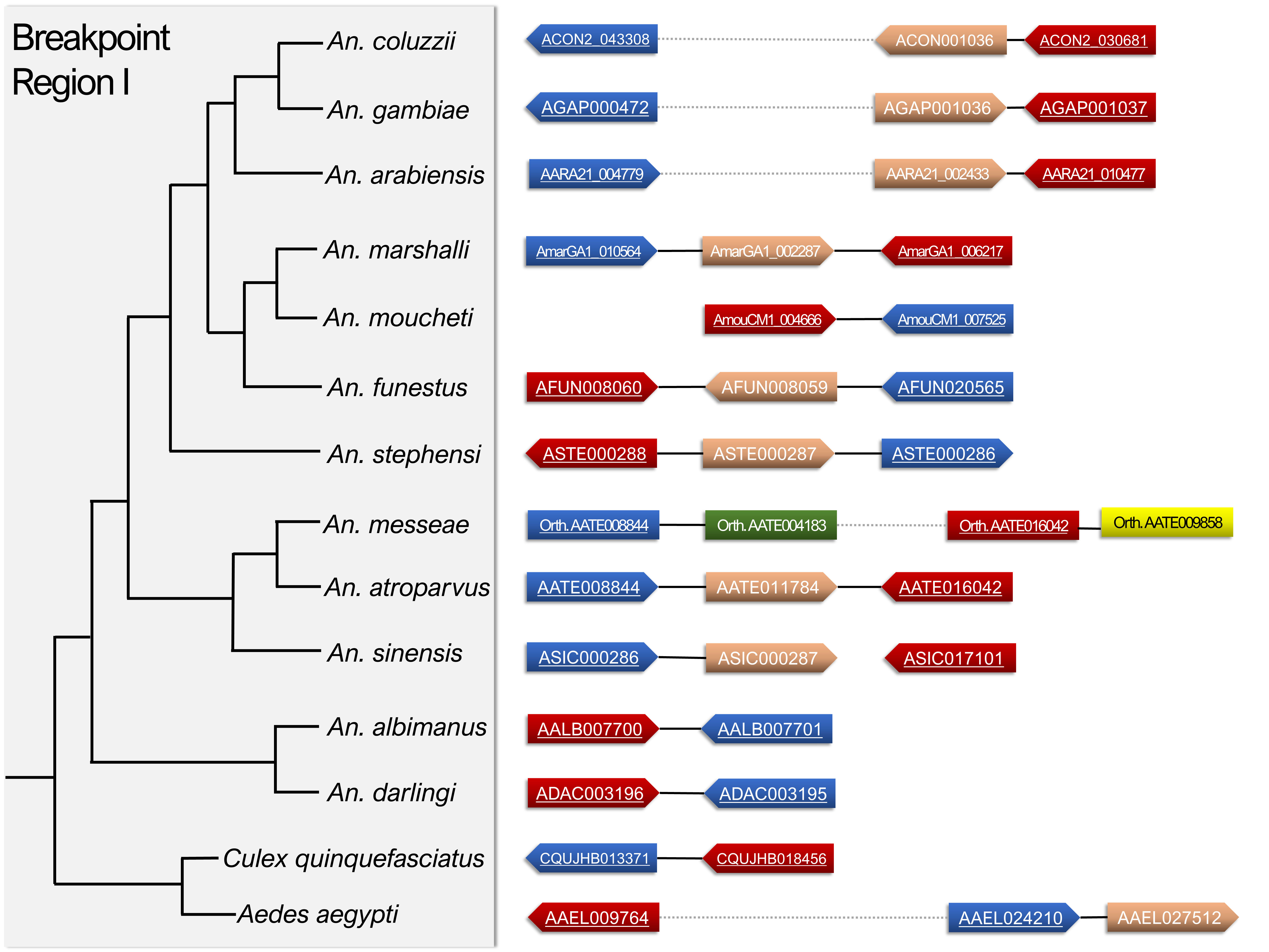

Supplement: Supplementary file 1 [file insects-15-00312-s001.zip › fig S1.tif]

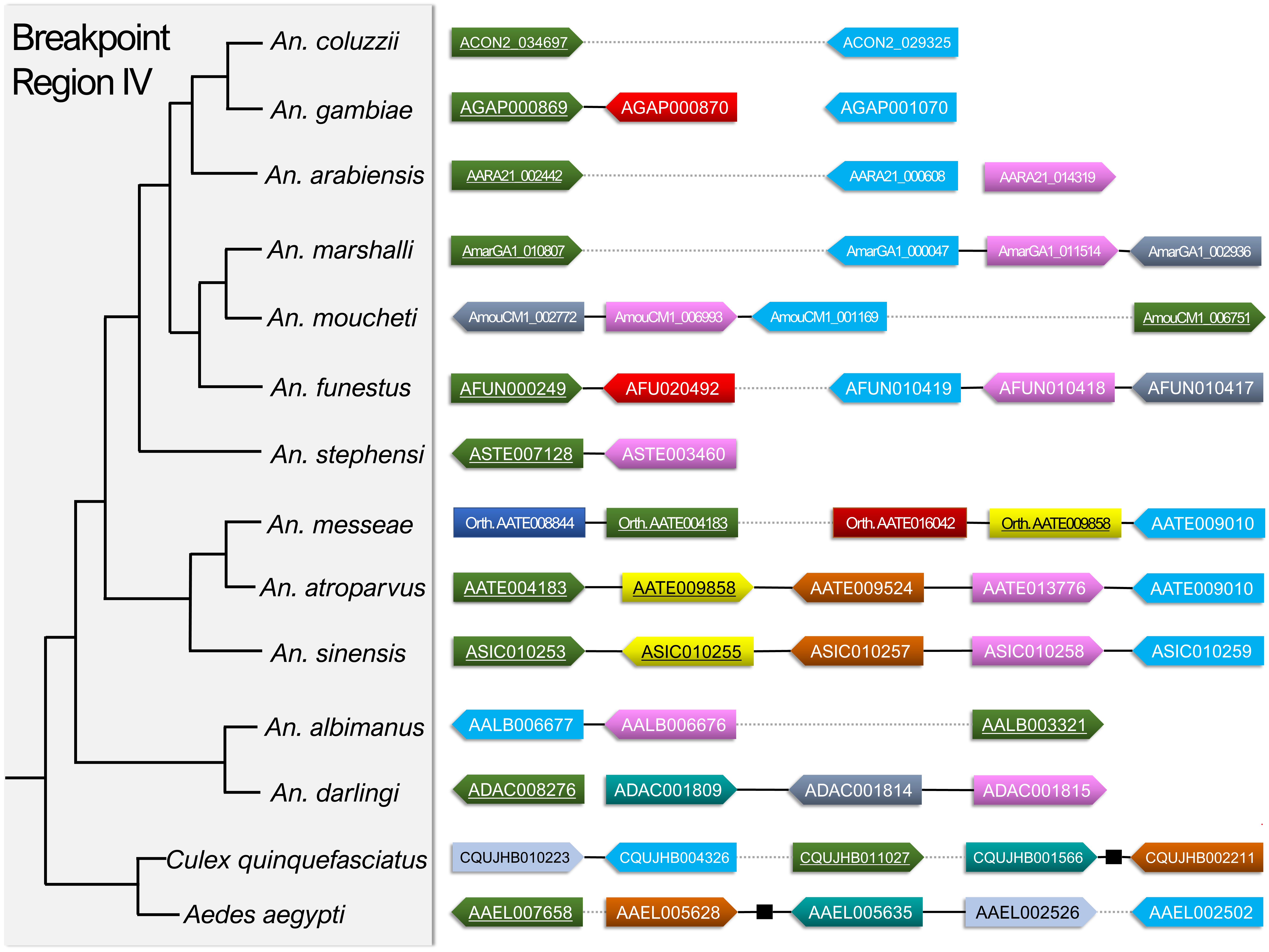

Supplement: Supplementary file 1 [file insects-15-00312-s001.zip › fig S2.tif]

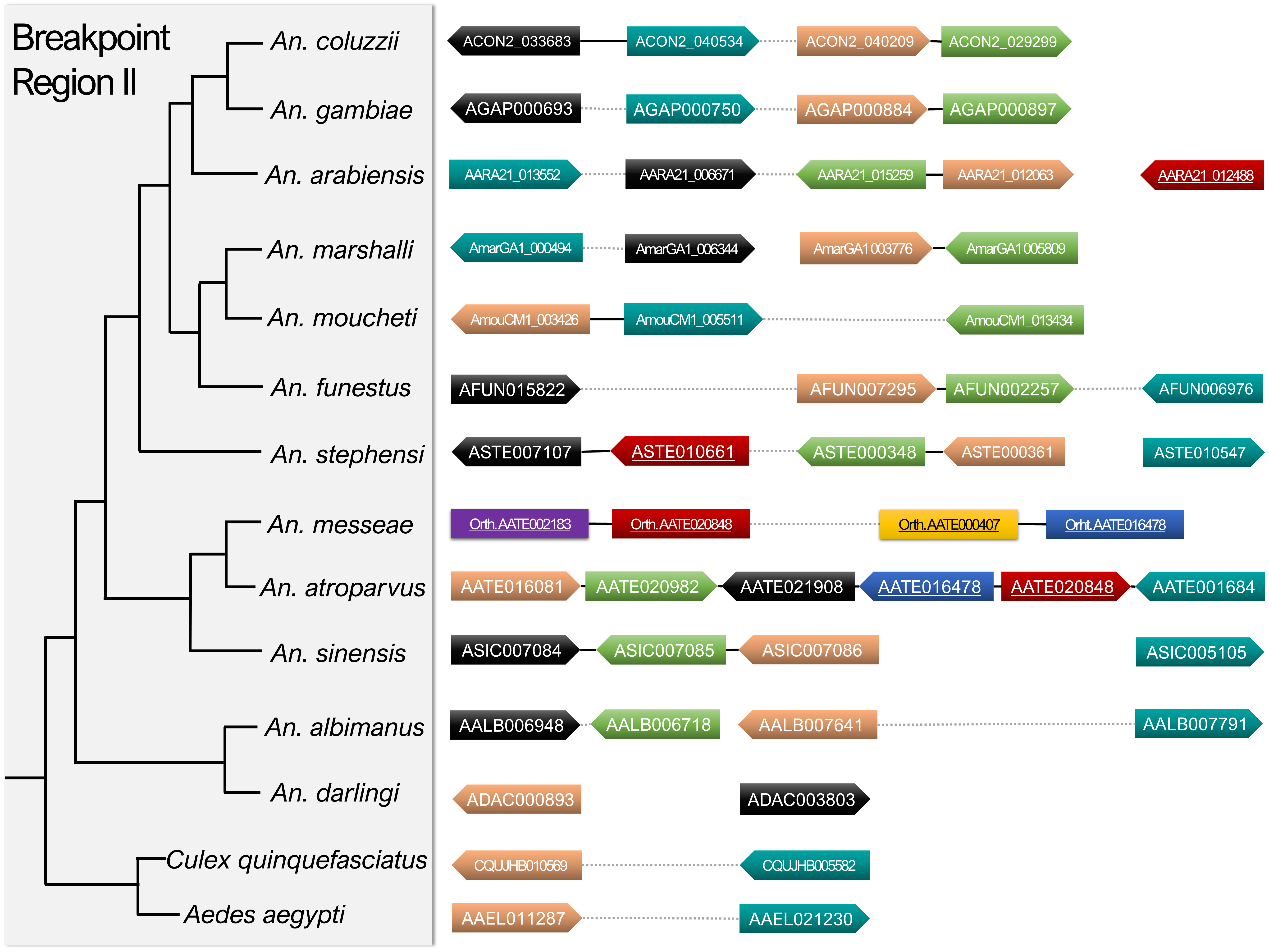

Supplement: Supplementary file 1 [file insects-15-00312-s001.zip › fig S3.tif]

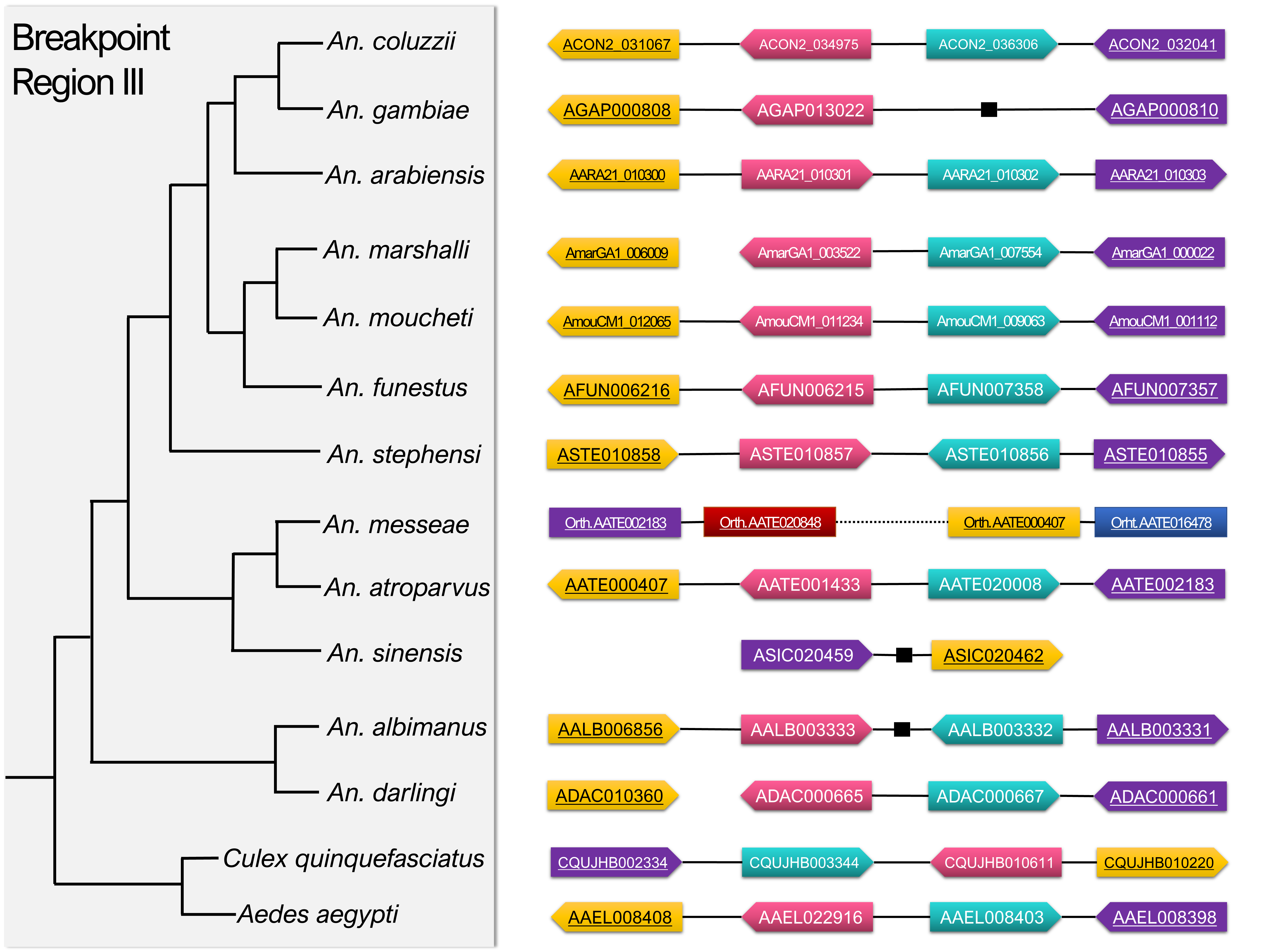

Supplement: Supplementary file 1 [file insects-15-00312-s001.zip › fig S4.tif]

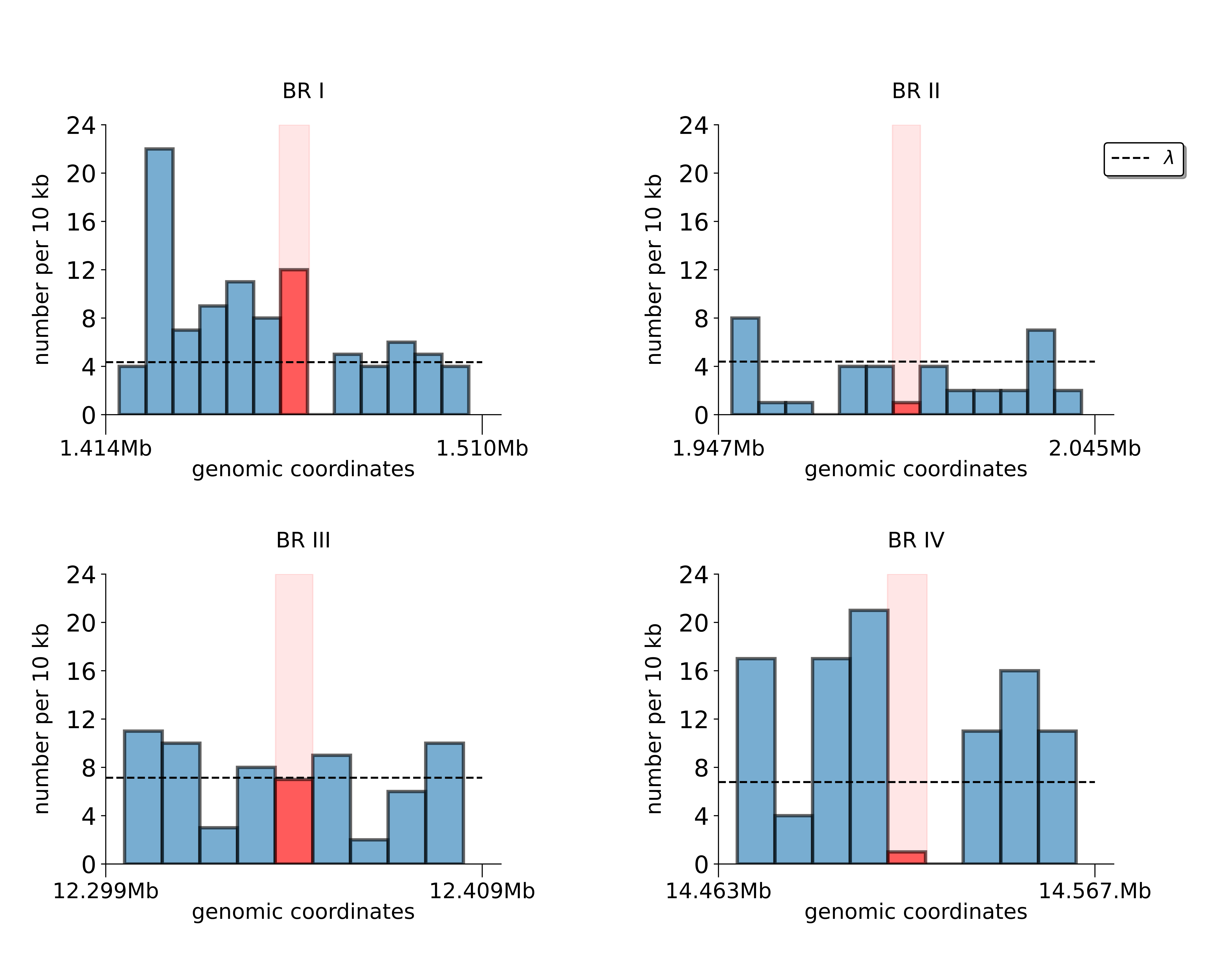

Supplement: Supplementary file 1 [file insects-15-00312-s001.zip › fig S5.tif]

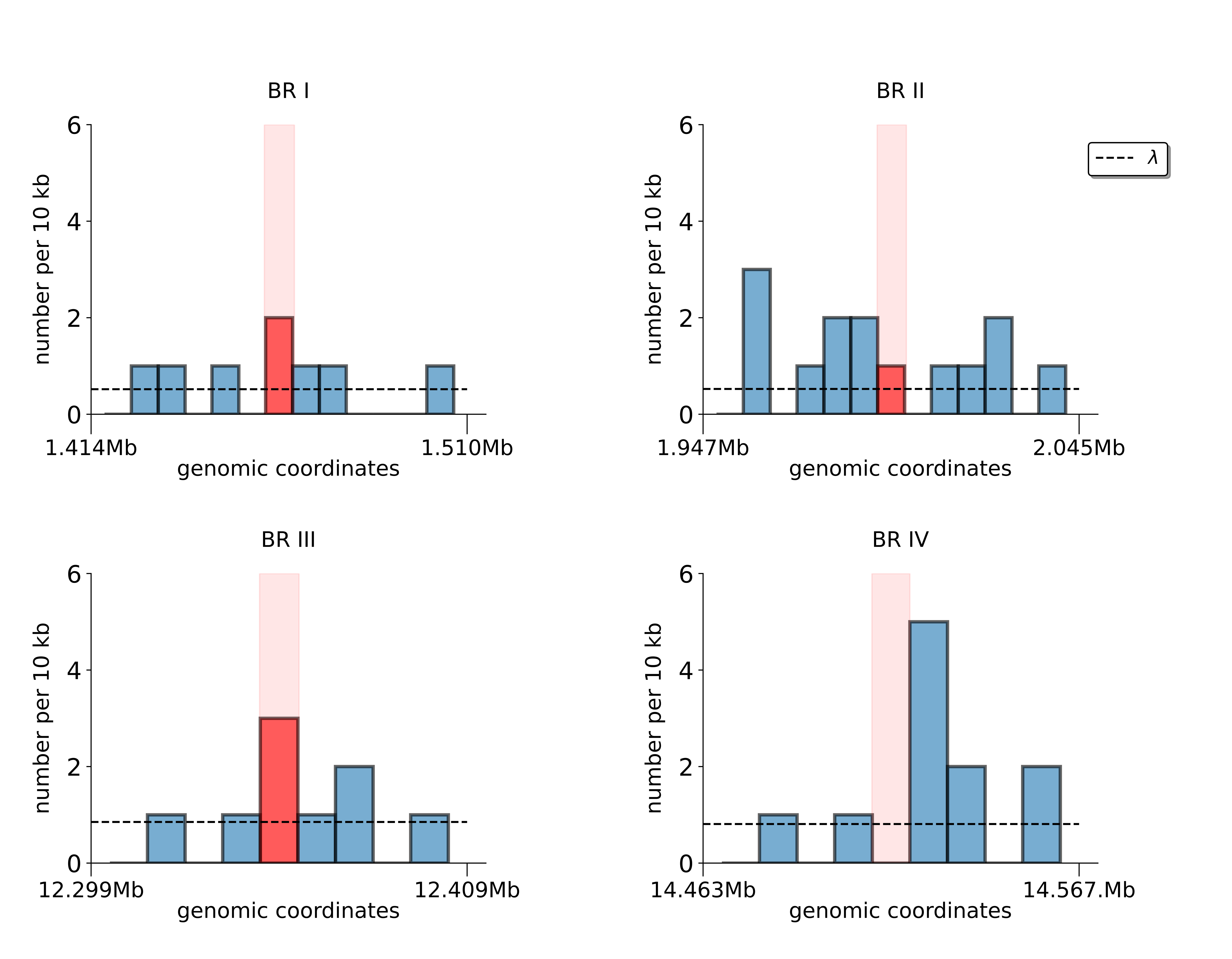

Supplement: Supplementary file 1 [file insects-15-00312-s001.zip › fig S6.tif]
